# Clinical Features and Prognoses of Patients With Breast Cancer Who Underwent Surgery

**DOI:** 10.1001/jamanetworkopen.2023.31078

**Published:** 2023-08-25

**Authors:** Gang Liu, Xiangyi Kong, Qichen Dai, Han Cheng, Jing Wang, Jidong Gao, Yipeng Wang

**Affiliations:** 1 Department of Breast Surgical Oncology, National Cancer Center/National Clinical Research Center for Cancer/Cancer Hospital, Chinese Academy of Medical Sciences and Peking Union Medical College, Beijing, China; 2 Department of Breast Surgical Oncology, National Cancer Center/National Clinical Research Center for Cancer/Cancer Hospital and Shenzhen Hospital, Chinese Academy of Medical Sciences and Peking Union Medical College, Shenzhen, China

## Abstract

**Importance:**

Breast cancer (BC) remains a pervasive malignant neoplasm worldwide, with increasing incidence. However, there are a scarcity of studies examining the clinical characteristics and prognosis of Chinese patients with BC who have undergone surgery.

**Objective:**

To evaluate overall survival (OS) and disease-free survival (DFS) in patients with surgically treated BC in China, focusing on histopathology and surgical approach.

**Design, Setting, and Participants:**

This cohort study included a retrospective review of the medical records of patients with unilateral BC who underwent surgery between January 2009 and September 2017, with a median follow-up time of 7.69 years. Clinical features were extracted from these records, and survival analysis was performed. Data analysis was conducted in March 2023.

**Main Outcomes and Measures:**

Patients’ OS and DFS.

**Results:**

The study included 14 782 patients (14 724 [99.6%] female patients; mean [SD] age, 51.6 [10.9] years). Invasive ductal carcinoma (IDC) was the most prevalent type, observed in 12 671 patients (85.6%). Stages 0, I, II, III, and IV accounted for 6.4% (919 patients), 32.0% (4579 patients), 40.5% (5791 patients), 20.2% (2896 patients), and 0.9% (126 patients) of cases, respectively. Hormone receptor (HR) positivity was observed in 10 241 patients (75.1%), and 3665 (29.1%) tested positive for *ERBB2* (formerly *HER2*/neu). The HR-negative–*ERBB2*-negative, HR-negative–*ERBB2*-positive, HR-positive–*ERBB2*-negative, and HR-positive–*ERBB2*-positive subtypes constituted 13.3% (1666 patients), 12.7% (1595 patients), 57.8% (7251 patients), and 16.2% (2034 patients) of cases, respectively. Breast-conserving surgery (BCS) was performed in 2884 patients (19.5%). The 5-year and 10-year OS rates were 92.9% (13 689 of 14 732) and 87.4% (3287 of 3760), while the 5-year and 10-year DFS rates were 89.0% (12 916 of 14 512) and 82.9% (3078 of 3713), respectively. Multivariate analysis found that for patients with IDC, age, BCS, invasive tumor size, tumor grade, lymphovascular invasion (LVI), the number of lymph node metastases (LNMs), distant metastasis, Ki67, and HR status were associated with OS, whereas invasive tumor size, tumor grade, LVI, the number of LNMs, HR status, and *ERBB2* status were associated with DFS. After propensity score matching, BCS was equivalent to mastectomy with respect to survival in patients with IDC.

**Conclusions and Relevance:**

This cohort study of patients with BC who underwent surgery in China provides valuable insights into the histopathological characteristics and survival outcomes of this population. The diverse histopathological features emphasize the necessity for customized treatment strategies. The relatively low BCS rate in the study population suggests the need for heightened awareness and adoption of this approach, considering its potential advantages for survival.

## Key Points

**Question:**

What are the clinical characteristics and prognosis of Chinese patients with breast cancer who have undergone surgery?

**Findings:**

This cohort study analyzed 14 782 patients with unilateral breast cancer who underwent surgery and found varied histopathological characteristics and survival outcomes. Less than one-fifth of patients underwent breast-conserving surgery, but that approach had at least equivalent survival to mastectomy.

**Meaning:**

The relatively low rate of breast-conserving surgery in the study population suggests the need for heightened awareness and adoption of this approach, considering its potential advantages for patient survival.

## Teaser

This cohort study evaluates overall and disease-free survival in patients with surgically treated breast cancer in China.

## Introduction

Breast cancer (BC) is the most frequently diagnosed cancer type and ranks among the top 5 leading causes of cancer-related deaths worldwide.^1^ In China, BC also has the highest incidence and is one of the most lethal cancers in women.^2^ It is widely acknowledged that BC is a heterogeneous disease that can be classified in various ways, including by histological type and molecular features. Literature reviews suggest that histological type plays a crucial role in distinguishing and predicting the prognosis of patients with BC.^3^ The most prevalent histological type is invasive ductal carcinoma (IDC), accounting for approximately 75% of all BC cases.^4^ Another common histological type with a favorable prognosis is ductal carcinoma in situ (DCIS).^5^ Other unique types exhibit distinct biological features and clinical behaviors. Prior studies have shown that the proportions of these unique histological types vary across races. In the United States, the White population has a higher proportion of IDC, while Black and Asian American populations have a greater percentage of special types.^6^ However, in China, most relevant studies have primarily compared only 1 or 2 special types individually,^7^ and research investigating the proportions and prognoses remains scarce.

Previous research has demonstrated that numerous factors are associated with BC prognosis. Not only do the histological type and molecular features influence survival, but the treatment type, such as the surgical approach, can also impact prognosis. As prognoses improve, an increasing number of patients are focusing on breast satisfaction and quality of life.^8^ According to a randomized clinical trial,^9^ breast-conserving surgery (BCS) yields similar prognoses to mastectomy in patients with early-stage BC.^9^ Recent studies have also provided evidence that BCS may offer better prognoses than mastectomy.^10,11^ However, in China, the BCS rate is low, and there are few studies comparing the prognoses of patients undergoing BCS vs mastectomy.^12,13^ It is essential to conduct research to determine the current status of BCS and its influence on survival in China.

In our study, we detailed the clinical and histopathological features of patients with BC who underwent surgery in a medical center of China from 2009 to 2017 and analyzed overall survival (OS) and disease-free survival (DFS) in all patients. Additionally, we compared the prognoses of patients who underwent BCS and mastectomy to provide an overview of the surgical treatment landscape for BC in China.

## Methods

### Patients and Materials

In this study, we reviewed the medical records of patients with unilateral BC who underwent surgery at the Cancer Hospital of the Chinese Academy of Medical Sciences (CHCAMS) from January 2009 to September 2017. The CHCAMS is a renowned medical institution in China specializing in the diagnosis, treatment, and research of cancer. Located in Beijing, it is affiliated with the Chinese Academy of Medical Sciences and serves as a national center for cancer prevention and control. The medical data were obtained from the electronic medical record. The cohort was carefully documented in a manner similar to our local tumor registry. Our team, comprised of approximately 10 individuals, was responsible for manually extracting the data. The medical records included data on age, sex, distant metastasis (DM), surgical strategy (BCS or mastectomy), and histopathological features, such as invasive tumor size, number of lymph node metastases (LNMs), tumor grade, Ki67, lymphovascular invasion (LVI), nerve invasion (NI), hormone receptor (HR) status, and *ERBB2* (formerly *HER2*/neu) status. BCS includes patients who have received BCS, whether or not they completed adjuvant radiation. Tumor specimens were graded histopathologically based on the fifth World Health Organization classification and staged according to the eighth American Joint Committee on Cancer (AJCC) TNM staging system. For patients with mixed histopathological types, the invasive histopathology type with the higher proportion was recorded. For patients who underwent neoadjuvant chemotherapy (NAC), the clinical staging system was used. Survival time was calculated from the date of diagnosis to either the date of event occurrence or the censoring date. Our study followed the Strengthening the Reporting of Observational Studies in Epidemiology (STROBE) reporting guideline. The study was approved, and a waiver of informed consent due to the retrospective nature of the study was granted by the Ethics Committee.

### Statistical Analysis

Data analysis was performed using SPSS software version 25.0 (IBM Corp). Differences between groups were evaluated using the χ^2^ test, Fisher test, or independent samples *t* test, depending on the characteristics of the data. Survival analysis was performed using Kaplan-Meier methods and an adjusted Cox proportional hazards model. For continuous variables, such as age and tumor size, 1 standard unit (1 year and 1 cm) was used as a increment to calculate the hazard ratios. To balance the differences between BCS and mastectomy in patients with IDC, propensity-score matching (PSM) was used with a ratio of 1:2. Potential prognostic factors with a *P* < .15 identified in the univariate survival analysis were selected for the multivariable Cox proportional hazards regression analysis and to calculate the propensity scores. Statistical significance was defined as *P* < .05. Data analysis was conducted in March 2023.

## Results

### Basic Clinical and Pathological Features

A total of 14 782 individuals with unilateral BC who underwent surgery were included. The mean (SD) age of the patients was 51.63 (10.94) years, and the median age was 51 years, with a range from 19 to 94 years old (Table 1). Nearly all of the patients were female (14 724 [99.6%]). The most common histopathological type was IDC, accounting for 85.6% of cases (12 671 patients). Other types observed included DCIS, microinvasive BC (MIBC), mucinous carcinoma (MucC), invasive lobular carcinoma (ILC), medullary carcinoma (MedC), invasive micropapillary carcinoma (IMPC), metaplastic carcinoma (MPC), and apocrine carcinoma (AC). Additionally, 0.8% of cases (114 patients) were classified as other types of invasive BC (OTIBC), which encompassed occult BC, invasive papillary carcinoma, cribriform carcinoma, tubular carcinoma, adenoid cystic carcinoma, malignant phyllodes tumor, carcinoma with neuroendocrine features, and other infrequent subtypes.

**Table 1.** Characteristics of Patients With Breast Cancer Who Underwent Surgery

Characteristic	Patients by subtype, No. (%)										
	IDC	DCIS	MIBC	MucC	ILC	MedC	IMPC	MPC	AC	OTIBC	Total
Total No.	12 671 (85.6)	945 (6.3)	427 (2.9)	212 (1.5)	202 (1.4)	62 (0.4)	64 (0.4)	38 (0.3)	47 (0.4)	114 (0.8)	14 782 (100.0)
Sex											
Female	12 624 (99.6)	939 (99.4)	425 (99.5)	212 (100)	202 (100)	62 (100)	64 (100)	38 (100)	47 (100)	111 (97.4)^a^	14 724 (99.6)
Male	47 (0.4)	6 (0.6)	2 (0.5)	0	0	0	0	0	0	3 (2.6)^a^	58 (0.4)
Age, mean (SD), y	51.56 (10.88)	50.64 (10.87)^a^	51.65 (10.18)	55.44 (14.42)^a^	53.28 (10.24)^a^	49.55 (11.94)	52.09 (10.40)	55.08 (11.53)^a^	59.40 (9.67)^a^	54.05 (11.73)^a^	51.63 (10.94)
Concurrent DCIS	5482 (43.3)	945 (100)^a^	401 (93.9)^a^	57 (26.9)^a^	82 (40.6)^a^	7 (11.3)^a^	28 (43.8)	13 (34.2)	21 (44.7)	32 (28.1)^a^	7068 (47.8)
LVI	2485 (19.6)	0^a^	9 (2.1)^a^	12 (5.7)^a^	11 (5.4)^a^	1 (1.6)^a^	32 (50.0)^a^	2 (5.3)	4 (8.5)	9 (7.9)	2565 (17.4)
NI	1076 (8.5)	3 (0.3)^a^	0^a^	2 (0.9)^a^	38 (18.8)^a^	2 (3.2)	5 (7.8)	1 (2.6)	4 (8.5)	3 (2.6)	1134 (7.7)
Stage											
0	0	917 (97.0)	0	0	0	0	0	0	0	2 (2.4)	919 (6.4)
I	3958 (32.1)	0	359 (95.2)	93 (50.8)	62 (32.3)	28 (50.9)	16 (26.7)	10 (31.3)	18 (41.9)	35 (41.2)	4579 (32.0)
II	5491 (44.5)	20 (2.1)	14 (3.7)	78 (42.6)	79 (41.1)	27 (49.1)	9 (15.0)	18 (56.3)	21 (48.8)	34 (40.0)	5791 (40.5)
III	2773 (22.5)	7 (0.8)	4 (1.1)	11 (6.0)	48 (25.0)	0	34 (56.7)	3 (9.4)	4 (9.3)	12 (14.1)	2896 (20.2)
IV	117 (0.9)	1 (0.1)	0	1 (0.6)	3 (1.6)	0	1 (1.7)	1 (3.1)	0	2 (2.4)	126 (0.9)
HR positive	9057 (75.7)	464 (73.0)	236 (60.5)^a^	178 (97.3)^a^	177 (93.7)^a^	15 (27.3)^a^	45 (77.6)	5 (14.7)^a^	3 (7.0)^a^	61 (73.5)	10 241 (75.1)
*ERBB2* positive	3145 (28.0)	243 (52.9)^a^	200 (61.7)^a^	15 (8.8)^a^	11 (5.9)^a^	12 (22.6)	28 (49.1)^a^	2 (6.3)	1 (2.6)^a^	8 (10.0)^a^	3665 (29.1)
HR-negative and ERBB2-negative	1497 (13.4)	23 (5.1)^a^	21 (6.5)^a^	3 (1.8)^a^	10 (5.4)	31 (58.5)^a^	3 (5.4)	25 (78.1)^a^	35 (89.7)^a^	18 (22.8)^a^	1666 (13.3)
HR-negative and *ERBB2*-positive	1320 (11.8)	130 (28.8)^a^	118 (36.5)^a^	1 (0.6)^a^	2 (1.1)^a^	8 (15.1)	10 (17.9)	2 (6.3)	1 (2.6)	3 (3.8)	1595 (12.7)
HR-positive and *ERBB2*-negative	6548 (58.7)	189 (41.8)^a^	103 (31.9)^a^	150 (89.3)^a^	164 (88.6)^a^	10 (18.9)^a^	26 (46.4)	5 (15.6)^a^	3 (7.7)^a^	53 (67.1)	7251 (57.8)
HR-positive and ERBB2-positive	1794 (16.1)	110 (24.3)^a^	81 (25.1)^a^	14 (8.3)	9 (4.9)^a^	4 (7.5)	17 (30.4)	0	0	5 (6.3)	2034 (16.2)
NAC	1382 (10.9)	6 (0.6)^a^	0^a^	3 (1.4)^a^	30 (14.9)	2 (3.2)	15 (23.4)	3 (7.9)	1 (2.1)	11 (9.6)	1453 (9.8)
Underwent BCS	2328 (18.4)	270 (28.6)^a^	87 (20.4)	64 (30.2)^a^	34 (16.8)	21 (33.9)	8 (12.5)	12 (31.6)	16 (34.0)	44 (38.6)^a^	2884 (19.5)
Survival, %											
5-y OS	92.2	98.7	99.1	98.1	92.5	98.4	92.2	78.9	97.9	92.9	92.9
10-y OS	86.2	96.7	97.5	93.5	86.0	96.3	79.4	71.8	95.1	86.4	87.4
5-y DFS	87.8	97.7	97.7	96.2	91.4	98.4	87.0	83.6	97.9	93.6	89.0
10-y DFS	81.5	93.9	94.4	93.3	83.6	95.4	74.0	83.6	82.4	78.5	82.9

Abbreviations: AC, apocrine carcinoma; BCS, breast-conserving surgery; DCIS, ductal carcinoma in situ; DFS, disease-free survival; HR, hormone receptor; IDC, invasive ductal carcinoma; ILC, invasive lobular carcinoma; IMPC, invasive micropapillary carcinoma; LVI, lymphovascular invasion; MedC, medullary carcinoma; MIBC, microinvasive breast cancer; MPC, metaplastic carcinoma; MucC, mucinous carcinoma; NAC, neoadjuvant chemotherapy; NI, nerve invasion; OS, overall survival; OTIBC, other types of invasive breast cancer.

^a^ Had significant difference compared with IDC at *P* < .05 level.

Nearly half of the patients (7068 [47.8%]) presented with DCIS, while 2565 (17.4%) had LVI and 1134 (7.7%) had NI. The TNM staging system was used to classify patients as having stage 0 (919 [6.4%]), stage I (4579 [32.0%]), stage II (5791 [40.5%]), stage III (2896 [20.2%]), and stage IV (126 [0.9%]). Among all patients, 10 241 (75.1%) had HR-positive disease, and 3665 (29.1%) had *ERBB2*-positive disease. Patients were further categorized based on HR and *ERBB2* status, with 57.8% (7251) being HR-positive–*ERBB2*-negative. The HR-negative–ERBB2-negative, HR-negative–ERBB2-positive, and HR-positive–ERBB2-positive subtypes constituted 13.3% (1666 patients), 12.7% (1595 patients), and 16.2% (2034 patients) of cases, respectively. NAC was administered to 1453 patients (9.8%), and BCS was performed for 2884 patients (19.5%).

In comparison with the mean age of patients with IDC (51.56 [10.88] years), the mean age of patients with DCIS (50.64 [10.87] years) (*P* = .01) were significantly younger, while the mean ages of patients with MucC (55.44 [14.42] years) (*P* < .001), ILC (53.28 [10.24] years) (*P* = .03), MPC (55.08 [11.53] years; (*P* = .046), and AC (59.40 [9.67] years) (*P* < .001) were significantly older. The proportion of patients exhibiting concurrent DCIS was approximately 43.3% (5482) among patients with IDC. This percentage was higher in patients with MIBC (401 [93.9%]) and lower in patients with MucC (57 [26.9%]), ILC (82 [40.6%]), and MedC (7 [11.3%]).

Approximately 9057 patients with IDC (75.7%) had HR-positive disease, and 3145 (28.0%) had *ERBB2*-positive disease. The percentage of patients with HR positivity was significantly lower in the MIBC (236 [60.5%]) (*P* < .001), MedC (15 [27.3%]) (*P* < .001), MPC (5 [14.7%]) (*P* < .001), and AC (3 [7.0%]) (*P* < .001) groups than in the IDC group. Conversely, the percentage of patients with HR positivity was significantly higher in the MucC (178 [97.3%]) (*P* < .001) and ILC (177 [93.7%]) (*P* < .001) groups.

The percentage of patients with *ERBB2* positivity was significantly lower in the MucC (15 [8.8%]) (*P* < .001), ILC (11 [5.9%]) (*P* < .001), and AC (1 [2.6%]) (*P* < .001) groups than in the IDC group. In contrast, the percentage of patients with *ERBB2* positivity was significantly higher in the DCIS (243 [52.9%]) (*P* < .001), MIBC (200 [61.7%]) (*P* < .001), and IMPC (28 [49.1%]) (*P* < .001) groups. The percentages of patients with HR-negative–*ERBB2*-positive, *HR*-negative–*ERBB2*-positive, HR-positive–*ERBB2*-negative, and HR-positive–*ERBB2*-positive subtypes in different histological types were also divergent (Table 1).

Of all patients with IDC, 1382 (10.9%) received NAC. The percentage of patients who received NAC was significantly lower in the DCIS (6 [0.6%]) (*P* < .001), MIBC (0) (*P* < .001), and MucC (3 [1.4%]) (*P* < .001) groups than in the IDC group. The proportion of patients who received BCS plus radiation was 92.3% (2662 patients). The rate of BCS in the IDC group was 18.4% (2328 patients), which was significantly lower than that in the DCIS (28.6% [270 patients]) (*P* < .001) and MucC (30.2% [64 patients]) (*P* < .001) groups.

### Survival Analysis of Different Histopathology Types

**Figure 1.**
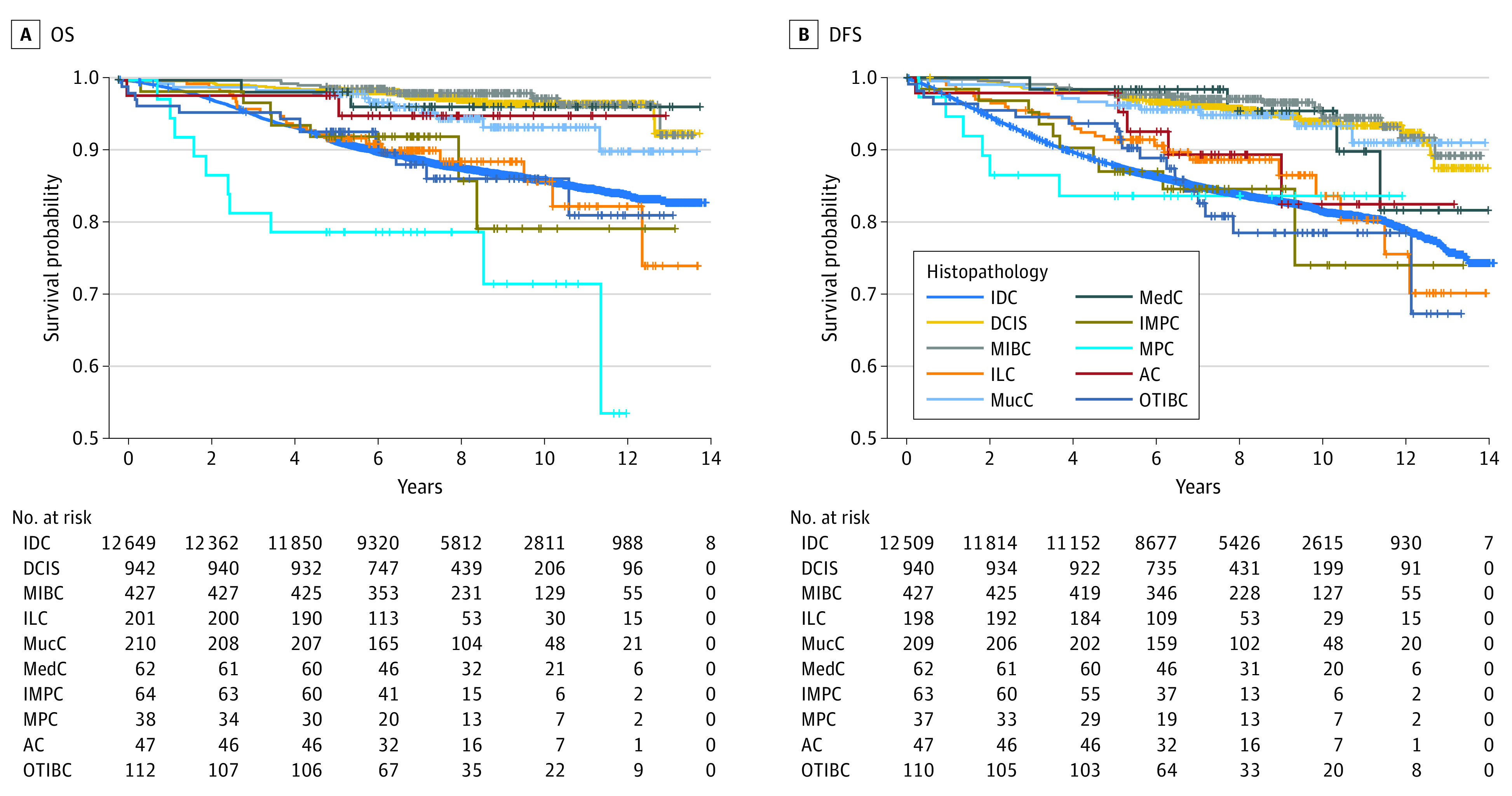
Survival Plots of Different Histopathological Types Using the Univariate Kaplan-Meier Method AC indicates apocrine carcinoma; DCIS, ductal carcinoma in situ; DFS, disease-free survival; IDC, invasive ductal carcinoma; ILC, invasive lobular carcinoma; IMPC, invasive micropapillary carcinoma; MedC, medullary carcinoma; MIBC, microinvasive breast cancer; MPC, metaplastic carcinoma; MucC, mucinous carcinoma; OTIBC, other types of invasive breast cancer; and OS, overall survival.

The median (IQR) follow-up time was 7.69 (5.86-9.72) years. In our study, there were 1622 deaths and 2246 cases of disease progression. The rate of loss to follow-up was 0.2% (30 patients) for OS and 0.8% (114 patients) for DFS. The 5-year and 10-year OS rates were 92.9% (13 689 of 14 732) and 87.4% (3287 of 3760), and the 5-year and 10-year DFS rates were 89.0% (12 916 of 14 512) and 82.9% (3078 of 3713), respectively (Table 1). Survival analysis (eTable 1 in Supplement 1) revealed that DCIS, MIBC, and MucC were associated with better OS and MPC was associated with worse OS compared with IDC (Figure 1A). We also found that compared with IDC, DCIS, MIBC, MucC, and MedC were associated with better DFS (Figure 1B).

To identify the factors that were associated with survival, we conducted univariate survival analysis using the Cox regression model (eTables 2 and 3 in Supplement 1). Factors such as age, breast surgery type, concurrent DCIS, TNM stage, tumor grade, LVI, NI, HR status, and *ERBB2* status were found to be associated with survival in patients with different histopathological types. Upon performing multivariate survival analysis using the Cox regression model (eTable 4 in Supplement 1), we found that BCS and HR-positive status were associated with better OS. Age at diagnosis, MPC, invasive tumor size, grade 2/3 tumors (compared with grade 1 tumors), LVI, the number of LNMs, DM, and Ki67 were associated with worse OS. According to the analysis of DFS, HR-positive status was also associated with better outcomes. Furthermore, invasive tumor size, grade 2/3 tumors (compared with grade 1 tumors), LVI, and the number of LNM were associated with worse DFS.

### Survival Analysis of IDC

**Figure 2.**
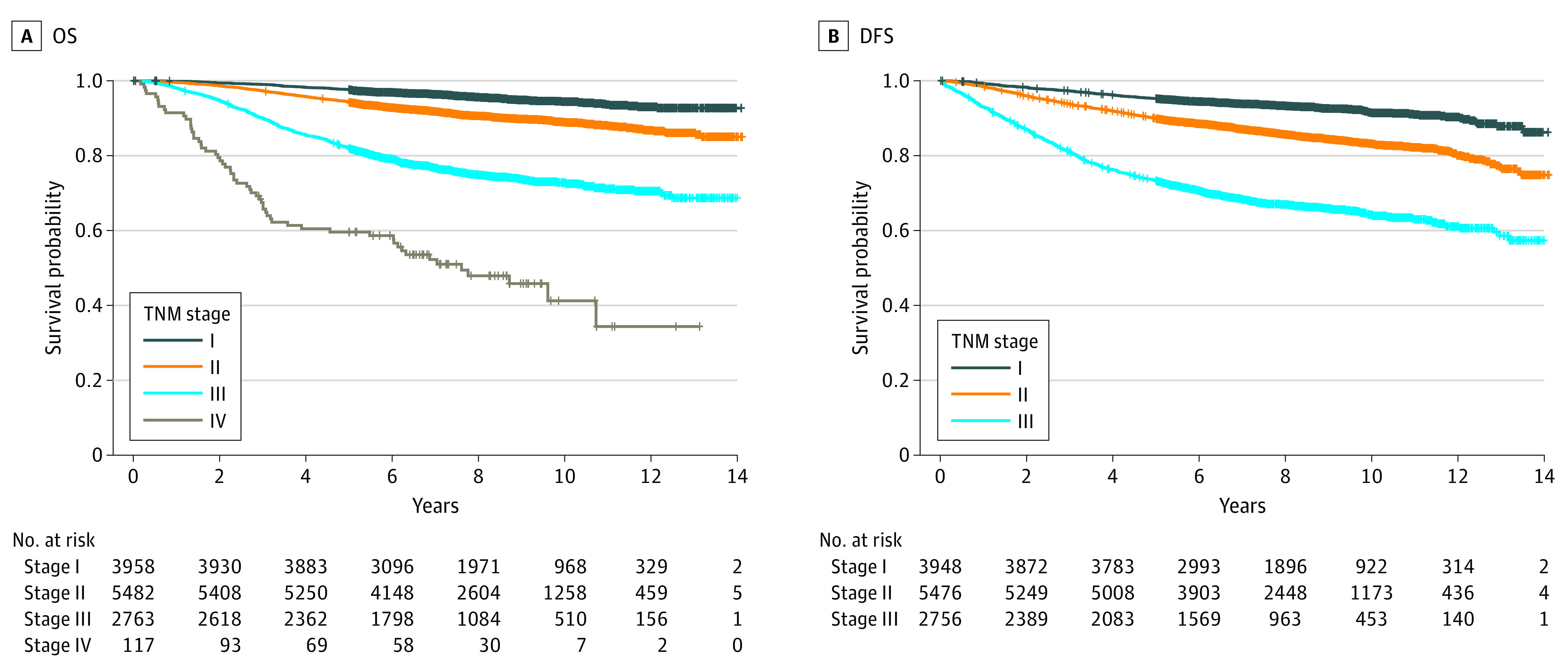
Stage-Related Survival Analysis of Patients With Invasive Ductal Carcinoma Using the Univariate Kaplan-Meier Method DFS indicates disease-free survival; OS, overall survival.

As patients with IDC accounted for 85.6% of all patients who underwent surgery (12 671 individuals), we conducted survival analysis specifically for patients with IDC. Stage-related OS and DFS curves are shown in Figure 2. For patients with stage I disease, the 5-year and 10-year OS rates were 97.7% (3860 of 3951) and 94.4% (968 of 1025), respectively, while the 5-year and 10-year DFS rates were 95.3% (3755 of 3940) and 91.5% (922 of 1008). For patients with stage II disease, the 5-year and 10-year OS rates were 94.4% (5173 of 5480) and 88.9% (1258 of 1415), respectively, while the 5-year and 10-year DFS rates were 90.0% (4891 of 5434) and 83.2% (1173 of 1410). For patients with stage III disease, the 5-year and 10-year OS rates were 82.2% (2266 of 2757) and 72.8% (510 of 701), respectively, while the 5-year and 10-year DFS rates were 73.4% (1998 of 2722) and 64.2% (453 of 706). For patients with stage IV disease, the 5-year and 10-year OS rates were 59.6% (68 of 114) and 41.2% (7 of 17), respectively.

Using the Cox regression model for multivariate survival analysis (Table 2), age at diagnosis, invasive tumor size, grade 2/3 tumors (compared with grade 1 tumors), LVI, the number of LNMs, DM, and Ki67 were associated with worse OS. Conversely, BCS (compared with mastectomy) and HR-positive status were associated with better OS. Moreover, invasive tumor size, grades 2 and 3 tumors (compared with grade 1 tumors), LVI, and the number of LNMs were also associated with worse DFS, while HR-positive and *ERBB2*-positive statuses were associated with better DFS. To evaluate the efficacy of BCS and mastectomy, we selected patients with T1/T2 disease without DM for PSM (eTable 5 in Supplement 1). After PSM, we found there was no significant difference of OS and DFS between patients who underwent BCS and mastectomy (eFigure in Supplement 1).

**Table 2.** Multivariate Analysis of Clinical and Pathological Factors Associated With OS and DFS in Patients With IDC

Factor	OS		DFS	
	Hazard ratio (95% CI)	*P* value	Hazard ratio (95% CI)	*P* value
Age	1.026 (1.020-1.032)	<.001	1.002 (0.997-1.007)	.49
BCS vs mastectomy	0.80 (0.64-1.00)	.047	1.02 (0.87-1.20)	.81
Concurrent DCIS	1.00 (0.86-1.12)	.76	0.99 (0.89-1.10)	.81
Invasive size	1.28 (1.23-1.34)	<.001	1.26 (1.21-1.30)	<.001
Grade 2 vs 1	1.89 (1.22-2.94)	.005	3.00 (1.99-4.52)	<.001
Grade 3 vs 1	2.26 (1.43-3.58)	<.001	3.17 (2.07-4.83)	<.001
LVI	1.33 (1.15-1.54)	<.001	1.36 (1.20-1.53)	<.001
NI	1.07 (0.86-1.33)	.54	1.05 (0.87-1.27)	.59
No. of LNMs	1.051 (1.045-1.058)	<.001	1.047 (1.042-1.053)	<.001
DM	4.31 (2.92-6.37)	<.001	NA	NA
Ki67	1.69 (1.21-2.36)	.002	1.16 (0.87-1.56)	.31
HR positive vs negative	0.66 (0.57-0.77)	<.001	0.82 (0.72-0.94)	.003
*ERBB2* positive vs negative	0.88 (0.76-1.01)	.07	0.88 (0.78-0.99)	.03

Abbreviations: BCS, breast-conserving surgery; DCIS, ductal carcinoma in situ; DFS, disease-free survival; DM, distant metastasis; HR, hormone receptor; IDC, invasive ductal carcinoma; LVI, lymphovascular invasion; LNM, lymph node metastases; NA, not applicable; NI, nerve invasion; OS, overall survival.

## Discussion

This study sought to delineate the clinical and pathological characteristics of patients with BC who underwent surgery in the CHCAMS between 2009 and 2017 while analyzing OS and DFS among all patients. The predominant patient population presented with IDC. Certain histopathological features were found to be associated with survival outcomes. BCS rates were comparatively low; however, patients who underwent BCS had potentially superior OS compared with those who underwent mastectomy.

One of the initial objectives of this study was to determine the proportions and features of different histopathological types. Consistent with previous literature, we found that most patients had IDC.^4,6^ Survival analysis showed that patients with DCIS and MIBC had better prognoses. Our results are consistent with previous studies that have shown that patients with MucC have a better prognosis than those with IDC,^14,15^ while patients with MedC have better DFS^16,17^ and those with MPC have worse OS.^18^

Our analysis revealed that the mean age of patients with BC who underwent surgery in our study was 51.63 years, which was older than that reported in previous studies^13,19^ but younger than the reported peak age range of 55 to 60 years in 2020.^20^ One possible explanation for this might be the increasing trend of age-standardized incidence rates.^20^ Consistent with prior research, our study showed that the mean age of patients with DCIS was younger, while the mean age of patients with MucC, ILC, MPC, and AC was significantly older than that of patients with IDC.^14,18,21,22,23^ We also found that age was associated with worse OS. However, the association between age and DFS in our study was not significant. Some studies have indicated that young age is associated with worse DFS.^24,25^ This inconsistency may be due to differences in histopathological types and stages. Further research should be conducted to investigate these differences.

A salient observation within our study was that nearly half of patients with IDC exhibited concomitant DCIS. In contrast, patients with MucC, ILC, and MedC had lower incidences of concurrent DCIS than their counterparts with IDC. Prior research has indicated that the prevalence of coexisting DCIS can range from 20.6% to 60.4% among distinct ethnic groups.^26,27^ In our study, univariate survival analysis suggested that the presence of concurrent DCIS was associated with OS and DFS in patients with IDC. Nevertheless, the detected discrepancy in survival was not statistically significant in the multivariate analysis. Multiple studies have proposed that IDC with concurrent DCIS is associated with an improved prognosis across various molecular subtypes.^26,27^ It is conceivable that both molecular subtype and ethnicity may influence this divergence. Consequently, additional subgroup analyses that take these variables into account are warranted.

Our study revealed that patients with DCIS, MIBC, MucC, ILC, and MedC had lower percentages of LVI, while patients with IMPC had higher percentages. Meanwhile, we also found that patients with DCIS, MIBC, and MucC had lower percentages of NI, while patients with ILC had higher percentages. Interestingly, a few patients with DCIS were found to have NI or DM, which is similar to previous studies.^28,29^

Our research offers an exhaustive analysis of HR and *ERBB2* statuses, finding that their distributions diverge among patients with distinct histopathological types in comparison with patients with IDC. As anticipated, we observed HR status as significantly associated with OS and DFS. Contrary to our expectations, however, we identified *ERBB2*-positive status as independently associated with better DFS in patients with IDC, which contradicts prior studies linking *ERBB2* positivity to an adverse prognosis.^30^ One feasible rationale for this incongruity is the use of anti-*ERBB2* therapy.

The paramount clinical discovery in this study was the potential superior OS exhibited by patients who underwent BCS compared with those who underwent mastectomy. Despite developed countries boasting a substantially elevated BCS rate, China’s BCS rate remains comparatively low.^12,31,32^ Our investigation observed an average BCS rate of 19.5%, exhibiting considerable variation (12.5%-38.6%) across distinct pathological classifications. This discrepancy can be partially attributed to a multitude of factors, including age at diagnosis, confidence in surgical practitioners, BCS endorsement, and financial insurance coverage.^33,34^ Notwithstanding the disparities in BCS rates, patients who underwent BCS had more favorable OS than their counterparts who underwent mastectomy, aligning with the conclusions of prior observational research.^10,35^ The precise mechanism underlying the OS enhancement in patients undergoing BCS remains elusive. One plausible hypothesis pertains to the implementation of adjuvant radiotherapy in patients following BCS, compared with patients without LNMs who undergo mastectomy and may not receive supplementary lymph node irradiation.^36^ Radiotherapy may effectively inhibit micrometastatic neoplastic cells, thereby augmenting OS.^37^ An alternative conjecture is that patients undergoing BCS typically have superior quality of life and reduced postoperative complications, particularly in the realms of psychosocial and sexual well-being.^8,38^ Preceding investigations have also suggested correlations among quality of life, mortality risk, and cancer-related outcomes.^39^ Another strength of our study is that we presented the association of BCS with both OS and DFS, whereas the National Cancer Database (NCDB) study from Wrubel et al^35^ only described OS. The fact that OS was superior for patients with BCS while DFS was unchanged could support the hypothesis that the protective association is not directly related to improved BC control and may come from reducing postsurgical complications or enhancing overall quality of life. The insights gleaned from these findings could potentially inform and guide surgical decision-making processes for both medical practitioners and patients.

### Limitations

Our study is subject to several limitations. First, as an observational investigation, this study is inherently prone to various confounding factors, such as missing data, which may impact the outcomes. Furthermore, the limited sample of patients with rare BC subtypes and the potential for censoring certain factors in the multivariate analysis could introduce bias in the survival analysis. Second, we did not examine clinical treatment information in the survival analysis. Despite conducting treatments based on the pathological findings for all patients, not every patient received standard care, which could introduce additional bias in the survival analysis. Third, we found superior OS among patients with IDC who underwent BCS in the multivariate survival analysis, but after PSM, we only found equivalent survival between patients who underwent BCS and mastectomy, possibly due to the low BCS rate and relatively small sample size. Further population-based research is needed for validation on a larger sample.

## Conclusions

In this cohort study of patients with BC who underwent surgery, we presented, to our knowledge, the most extensive analysis of survival in subgroups of patients with diverse histopathological classifications, encompassing a 10-year follow-up period in China. We elucidated the histopathological characteristics of these patients and the association between these characteristics and survival outcomes. Additionally, we found that patients who underwent BCS exhibited potentially superior OS, highlighting the need to augment BCS implementation in China.

## Notes

Supplement 1. **eTable 1.** Survival analysis of different pathological types in all breast cancer patients using the Cox regression model **eTable 2.** Univariate overall survival analysis of variables using Cox regression model **eTable 3.** Univariate disease-free survival analysis of variables using Cox regression model **eTable 4.** Multivariate analysis of clinical and pathologic factors associated with OS and DFS in all breast cancer patients **eTable 5.** Characteristics of T1-2 IDC patients who underwent surgery before and after propensity score matching **eFigure.** Survival plot of T1-2 IDC patients underwent BCS and mastectomy (M) in the propensity-score matching cohort

Supplement 2.
Data Sharing Statement
