# Supplementary material for: Clinical Features and Prognoses of Patients With Breast Cancer Who Underwent Surgery
Source: JAMA Netw Open. 2023 Aug 25;6(8):e2331078. doi: 10.1001/jamanetworkopen.2023.31078 (PMC10457722; doi:10.1001/jamanetworkopen.2023.31078)
Supplement: Supplement 1. — eTable 1. Survival analysis of different pathological types in all breast cancer patients using the Cox regression model eTable 2. Univariate overall survival analysis of variables using Cox regression model eTable 3. Univariate disease-free survival analysis of variables using Cox regression model eTable 4. Multivariate analysis of clinical and pathologic factors associated with OS and DFS in all breast cancer patients eTable 5. Characteristics of T1-2 IDC patients who underwent surgery before and after propensity score matching eFigure. Survival plot of T1-2 IDC patients underwent BCS and mastectomy (M) in the propensity-score matching cohort [file jamanetwopen-e2331078-s001.pdf]

Supplemental Online Content

Liu G, Kong X, Dai Q, et al. Clinical features and prognoses of patients with breast cancer who underwent surgery. *JAMA Netw Open.* 2023;6(8):e2331078. doi:10.1001/jamanetworkopen.2023.31078

eTable 1. Survival analysis of different pathological types in all breast cancer patients using the Cox regression model

eTable 2. Univariate overall survival analysis of variables using Cox regression model

eTable 3. Univariate disease-free survival analysis of variables using Cox regression model

eTable 4. Multivariate Analysis of Clinical and Pathologic Factors Associated With OS and DFS in all breast cancer patients

eTable 5. Characteristics of T1-2 IDC patients who underwent surgery before and after propensity score matching

eFigure. Survival plot of T1-2 IDC patients underwent BCS and mastectomy (M) in the propensity-score matching cohort

This supplemental material has been provided by the authors to give readers additional information about their work.

eTable 1. Survival analysis of different pathological types in all breast cancer patients using the Cox regression model.

	OS			DFS		
	p value	hazard ratio	95.0% CI	p value	hazard ratio	95.0% CI
DCIS vs IDC	**<0.001**	0.21	0.14-0.31	**<0.001**	0.28	0.21-0.37
MIBC vs IDC	**<0.001**	0.18	0.10-0.33	**<0.001**	0.24	0.15-0.37
MucC vs IDC	**0.004**	0.42	0.23-0.75	**<0.001**	0.32	0.18-0.57
ILC vs IDC	0.99	1.00	0.66-1.53	0.33	0.83	0.56-1.21
MedC vs IDC	0.05	0.25	0.06-1.02	**0.04**	0.35	0.13-0.94
IMPC vs IDC	0.98	1.01	0.48-2.13	0.88	1.05	0.56-1.95
MPC vs IDC	**0.002**	2.64	1.42-4.92	0.50	1.29	0.61-2.71
AC vs IDC	0.16	0.37	0.09-1.48	0.32	0.64	0.27-1.54
OTIBC vs IDC	0.90	1.04	0.60-1.79	0.96	1.01	0.64-1.61

Abbreviations: AC, apocrine carcinoma. DFS, disease-free survival. IDC, invasive ductal carcinoma. ILC, invasive lobular carcinoma. IMPC, invasive micropapillary carcinoma. MedC, medullary carcinoma. MIBC, microinvasive breast cancer. MPC, metaplastic carcinoma. MucC, mucinous carcinoma. OS, overall survival.

© 2023 Liu G et al. *JAMA Network Open.*

eTable 2. Univariate overall survival analysis of variables using Cox regression model.

	IDC		DCIS		MIBC		MucC		ILC		MedC		IMPC		MPC		AC		OTIBC	
	p value	hazard ratio (95% CI)	p value	hazard ratio (95% CI)	p value	hazard ratio (95% CI)	p value	hazard ratio (95% CI)	p value	hazard ratio (95% CI)	p value	hazard ratio (95% CI)	p value	hazard ratio (95% CI)	p value	hazard ratio (95% CI)	p value	hazard ratio (95% CI)	p value	hazard ratio (95% CI)
Female vs Male	0.33	0.71 (0.35-1.42)	0.80	20.28 (0.00-2.46*10^{11})	0.89	20.28 (0.00-8.61*10^{17})	NA	NA	NA	NA	NA	NA	NA	NA	NA	NA	NA	NA	0.74	20.77 (0.00-9.71*10^{8})
Age	<0.001	1.02 (1.02-1.03)	<0.001	1.06 (1.03-1.10)	<0.001	1.11 (1.06-1.17)	<0.001	1.09 (1.04-1.14)	0.002	1.06 (1.02-1.11)	0.06	1.09 (1.00-1.20)	1.00	1.00 (0.93-1.08)	0.10	1.04 (0.99-1.09)	0.25	1.08 (0.95-1.22)	0.22	1.03 (0.98-1.08)
BCS vs M	<0.001	0.70 (0.60-0.81)	0.52	1.31 (0.57-2.97)	0.06	3.27 (0.94-11.41)	0.27	1.96 (0.59-6.38)	0.13	0.21 (0.03-1.57)	0.57	0.03 (0.00-6.97*10^{3})	0.54	0.04 (0.00-1.20*10^{3})	0.12	0.20 (0.02-1.56)	0.43	142.31 (0.00-3.56*10^{7})	0.37	1.66 (0.55-4.95)
Concurrent DCIS	<0.001	0.78 (0.71-0.87)	NA	NA	0.58	22.31 (0.00-1.41*10^{6})	0.96	1.03 (0.27-3.89)	0.08	0.37 (0.12-1.11)	0.16	7.35 (0.46-117.58)	0.46	0.54 (0.10-2.78)	0.32	0.45 (0.10-2.14)	0.50	0.02 (0.00-1.90*10^{3})	0.25	0.41 (0.09-1.85)
Invasive Size	<0.001	1.38 (1.34-1.41)	NA	NA	NA	NA	0.05	1.23 (1.00-1.50)	<0.001	1.31 (1.13-1.52)	NA	NA	0.62	1.16 (0.64-2.11)	0.003	1.36 (1.11-1.67)	0.84	1.19 (0.23-6.10)	0.78	1.06 (0.72-1.56)
Grade 2 vs 1	<0.001	2.42 (1.76-3.34)	0.77	1.34 (0.19-9.50)	0.96	4.06*10^{4} (0.00-1.45*10^{196})	0.65	0.02 (0.00-7.65*10^{5})	0.97	2.69*10^{4} (0.00-8.84*10^{209})	NA	NA	1.00	1.02 (0.00-1.40*10^{23})	1.00	1.00 (0.00-3.61*10^{3})	0.77	65.27 (0.00-7.06*10^{13})	NA	NA
Grade 3 vs 1	<0.001	3.63 (2.63-5.02)	0.30	2.21 (0.49-9.89)	0.96	2.41*10^{4} (0.00-8.57*10^{195})	0.59	0.02 (0.00-4.61*10^{4})	0.97	3.05*10^{4} (0.00-1.00*10^{210})	0.86	22.30 (0.00-2.74*10^{16})	0.89	43.92 (0.00-2.43*10^{24})	1.00	1.00 (0.00-3.36*10^{3})	1.00	1.00 (0.00-5.03*10^{14})	0.40	2.57 (0.29-23.16)
LVI	<0.001	1.83 (1.64-2.05)	NA	NA	0.79	0.05 (0.00-3.54*10^{8})	0.60	0.05 (0.00-4.61*10^{3})	0.84	1.23 (0.16-9.22)	0.90	0.05 (0.00-3.72*10^{18})	0.37	2.13 (0.41-11.20)	0.03	5.85 (1.18-29.15)	0.76	0.04 (0.00-2.85*10^{7})	0.96	0.94 (0.12-7.33)
NI	0.08	1.17 (0.98-1.40)	0.85	0.05 (0.00-8.79*10^{11})	NA	NA	0.86	0.05 (0.00-5.70*10^{12})	0.39	0.53 (0.12-2.28)	0.88	0.05 (0.00-1.98*10^{15})	0.67	0.04 (0.00-6.37*10^{4})	0.05	8.74 (0.98-78.21)	0.78	0.04 (0.00-1.33*10^{8})	0.75	0.05 (0.00-5.21*10^{6})
Number Of LNM	<0.001	1.07 (1.06-1.07)	0.005	1.09 (1.02-1.15)	0.73	0.45 (0.00-46.58)	0.22	1.06 (0.97-1.16)	0.003	1.07 (1.02-1.12)	0.75	1.28 (0.27-6.02)	0.80	1.00 (0.95-1.07)	0.84	1.06 (0.60-1.86)	0.70	0.33 (0.00-96.05)	0.08	1.07 (0.99-1.15)
DM	<0.001	6.51 (5.02-8.42)	0.85	0.05 (0.00-3.63*10^{12})	NA	NA	0.88	0.05 (0.00-1.06*10^{15})	0.001	14.02 (3.14-62.61)	NA	NA	0.85	0.05 (0.00-1.52*10^{12})	0.75	0.05 (0.00-4.50*10^{6})	NA	NA	0.03	10.38 (1.26-85.41)
HR pos vs neg	<0.001	0.53 (0.48-0.59)	0.21	0.55 (0.21-1.41)	0.95	1.05 (0.29-3.75)	0.68	21.45 (0.00-5.22*10^{7})	0.01	0.24 (0.08-0.75)	0.69	0.03 (0.00-1.09*10^{6})	0.03	0.19 (0.04-0.84)	0.39	0.04 (0-71.27)	0.78	0.04 (0.00-1.76*10^{8})	0.01	0.17 (0.04-0.67)
HER2 pos vs neg	<0.001	1.28 (1.15-1.44)	0.02	5.56 (1.25-24.65)	0.88	0.89 (0.20-4.01)	0.54	0.04 (0.00-1.16*10^{3})	0.84	0.81 (0.11-6.07)	0.69	1.28*10^{3} (0.00-1.51*10^{18})	0.67	1.46 (0.25-8.42)	0.61	0.04 (0.00-6.90*10^{3})	0.91	0.05 (0.00-4.92-10^{22})	0.93	0.91 (0.11-7.32)

Abbreviations: AC, apocrine carcinoma. BCS, breast-conserving surgery. DCIS, ductal carcinoma in situ. DFS, disease-free survival. DM, distant metastasis. HER2, human epidermal growth factor receptor-2. HR, hormone receptor. IDC, invasive ductal carcinoma. ILC, invasive lobular carcinoma. IMPC, invasive micropapillary carcinoma. LNM, lymph node metastases. LVI, lymphovascular invasion. M, mastectomy. MedC, medullary carcinoma. MIBC, microinvasive breast cancer. MPC, metaplastic carcinoma. MucC, mucinous carcinoma. NA, not applicable. NI, nerve invasion. OTIBC, other types of invasive breast cancer.

© 2023 Liu G et al. *JAMA Network Open*.

eTable 3. Univariate disease-free survival analysis of variables using Cox regression model.

	IDC		DCIS		MIBC		MucC		ILC		MedC		IMPC		MPC		AC		OTIBC	
	p value	hazard ratio (95% CI)	p value	hazard ratio (95% CI)	p value	hazard ratio (95% CI)	p value	hazard ratio (95% CI)	p value	hazard ratio (95% CI)	p value	hazard ratio (95% CI)	p value	hazard ratio (95% CI)	p value	hazard ratio (95% CI)	p value	hazard ratio (95% CI)	p value	hazard ratio (95% CI)
Female vs male	0.38	0.76 (0.41-1.41)	0.73	20.28 (0.00-6.45*10^{8})	0.84	20.27 (0-7.65*10^{13})	NA	NA	NA	NA	NA	NA	NA	NA	NA	NA	NA	NA	0.23	3.50 (0.45-27.07)
Age	0.42	1.00 (0.99-1.00)	0.55	1.01 (0.98-1.03)	0.90	1.00 (0.96-1.05)	0.55	0.99 (0.95-1.03)	0.53	1.01 (0.98-1.05)	0.87	0.99 (0.91-1.09)	0.25	0.96 (0.91-1.03)	0.75	1.01 (0.95-1.08)	0.37	1.04 (0.95-1.14)	0.81	0.99 (0.95-1.04)
BCS vs M	<0.001	0.81 (0.71-0.91)	0.007	2.20 (1.24-3.91)	0.84	1.12 (0.40-3.13)	0.14	2.34 (0.75-7.25)	0.18	0.37 (0.09-1.57)	0.65	1.57 (0.22-11.29)	0.75	0.72 (0.09-5.66)	0.25	0.02 (0.00-13.46)	0.25	2.88 (0.48-17.25)	0.24	1.76 (0.69-4.51)
Concurrent DCIS	0.009	0.89 (0.82-0.97)	NA	NA	0.84	1.23 (0.16-9.18)	0.07	2.81 (0.90-8.71)	0.20	0.55 (0.22-1.38)	0.18	5.28 (0.47-59.60)	0.12	0.29 (0.06-1.36)	0.31	0.33 (0.04-2.83)	0.32	0.33 (0.04-2.95)	0.39	0.61 (0.20-1.87)
Invasive Size	<0.001	1.33 (1.30-1.36)	NA	NA	NA	NA	0.82	1.05 (0.67-1.66)	<0.001	1.52 (1.25-1.84)	0.98	1.02 (0.22-4.63)	0.22	1.31 (0.85-2.03)	0.08	1.23 (0.97-1.56)	0.09	2.90 (0.86-9.78)	0.77	1.05 (0.75-1.48)
Grade 2 vs 1	<0.001	3.45 (2.53-4.69)	0.09	3.28 (0.85-12.69)	0.96	1.05*10^{5} (0.00-4.19*10^{207})	NA	NA	0.96	2.32*10^{4} (0.00-3.60*10^{194})	NA	NA	NA	NA	1.00	1.00 (0.00-4.15*10^{3})	0.77	65.29 (0.00-7.06*10^{13})	0.96	9.38*10^{4} (0.00-3.05*10^{195})
Grade 3 vs 1	<0.001	4.27 (3.13-5.83)	0.06	3.19 (0.96-10.57)	0.96	6.63*10^{4} (0.00-2.64*10^{207})	0.34	3.30 (0.29-37.63)	0.96	5.55*10^{4} (0.00-8.61*10^{194})	0.81	24.64 (0.00-4.32*10^{12})	0.18	0.19 (0.02-2.20)	1.00	1.00 (0.00-3.48*10^{3})	1.00	1.00 (0.00-5.03*10^{14})	0.96	2.00*10^{5} (0.00-6.50*10^{195})
LVI	<0.001	1.82 (1.65-2.00)	NA	NA	0.73	0.05 (0.00-1.18*10^{6})	0.58	0.05 (0.00-2.91*10^{3})	0.17	2.76 (0.64-11.91)	0.93	0.05 (0.00-4.27*10^{28})	0.87	0.90 (0.26-3.16)	0.25	3.57 (0.41-30.72)	0.63	0.04 (0.00-1.85*10^{4})	0.74	1.29 (0.29-5.64)
NI	0.68	1.03 (0.88-1.21)	0.79	0.05 (0.00-2.65*10^{8})	NA	NA	0.89	0.05 (0.00-1.05*10^{18})	0.50	1.41 (0.52-3.85)	0.04	12.18 (1.07-137.98)	0.71	1.48 (0.18-11.81)	0.80	0.05 (0.00-7.11*10^{8})	0.65	0.04 (0.00-4.01*10^{4})	0.28	3.07 (0.40-23.47)
Number Of LNM	<0.001	1.06 (1.06-1.07)	0.01	1.08 (1.02-1.14)	0.89	1.03 (0.73-1.44)	0.37	1.04 (0.95-1.15)	<0.001	1.08 (1.03-1.12)	0.52	0.10 (0.00-109.80)	0.27	1.02 (0.98-1.06)	0.49	1.23 (0.68-2.21)	0.79	0.89 (0.37-2.11)	0.01	1.08 (1.02-1.15)
HR pos vs neg	<0.001	0.72 (0.65-0.79)	0.45	0.75 (0.36-1.59)	0.05	3.52 (1.03-12.11)	0.71	21.30 (0.00-1.65*10^{8})	0.03	0.30 (0.10-0.89)	0.97	1.05 (0.09-11.72)	0.004	0.16 (0.04-0.56)	0.49	0.04 (0.00-410.13)	0.63	0.04 (0.00-1.88*10^{4})	0.02	0.26 (0.09-0.79)
HER2 pos vs neg	0.02	1.13 (1.02-1.25)	0.06	2.42 (0.95-6.19)	0.18	0.49 (0.17-1.41)	0.50	0.04 (0.00-411.91)	0.18	2.30 (0.68-7.83)	0.89	1.49*10^{5} (0.00-1.76*10^{78})	0.22	2.34 (0.60-9.11)	0.66	0.04 (0.00-4.04*10^{4})	0.91	0.05 (0.00-4.92*10^{22})	0.73	0.69 (0.09-5.42)

© 2023 Liu G et al. *JAMA Network Open*.

Abbreviations: AC, apocrine carcinoma. BCS, breast-conserving surgery. DCIS, ductal carcinoma in situ. DFS, disease-free survival. DM, distant metastasis. HER2, human epidermal growth factor receptor-2. HR, hormone receptor. IDC, invasive ductal carcinoma. ILC, invasive lobular carcinoma. IMPC, invasive micropapillary carcinoma. LNM, lymph node metastases. LVI, lymphovascular invasion. M, mastectomy. MedC, medullary carcinoma. MIBC, microinvasive breast cancer. MPC, metaplastic carcinoma. MucC, mucinous carcinoma. NA, not applicable. NI, nerve invasion. OTIBC, other types of invasive breast cancer.

© 2023 Liu G et al. *JAMA Network Open*.

eTable 4. Multivariate Analysis of Clinical and Pathologic Factors Associated With OS and DFS in all breast cancer patients.

	OS			DFS		
	p value	hazard ratio	95.0% CI	p value	hazard ratio	95.0% CI
Age	**<0.001**	1.03	1.02-1.03	0.39	1.00	1.00-1.01
BCS vs M	**0.05**	0.80	0.64-1.00	0.74	1.03	0.88-1.21
OTIBC vs IDC	0.31	1.81	0.58-5.64	0.58	1.38	0.44-4.30
DCIS vs IDC	0.21	0.57	0.23-1.39	0.12	0.57	0.28-1.15
MIBC vs IDC	0.11	0.40	0.13-1.24	0.11	0.52	0.23-1.17
ILC vs IDC	0.59	1.37	0.44-4.26	0.61	1.29	0.48-3.46
MucC vs IDC	0.92	0	$0.00\text{-}5.87\times10^{64}$	0.65	1.58	0.22-11.33
MedC vs IDC	0.90	0	$0.00\text{-}2.59\times10^{56}$	0.55	0.55	0.08-3.92
IMPC vs IDC	0.40	0.43	0.06-3.08	0.22	0.29	0.04-2.07
MPC vs IDC	**0.03**	3.05	1.13-8.24	0.88	1.12	0.28-4.50
AC vs IDC	0.76	0.73	0.10-5.23	0.60	0.59	0.08-4.19
Concurrent DCIS	0.60	0.97	0.86-1.11	0.76	0.98	0.88-1.10
Invasive Size	**<0.001**	1.29	1.23-1.34	**<0.001**	1.26	1.21-1.30
Grade 2 vs 1	**0.007**	1.78	1.17-2.72	**<0.001**	2.91	1.96-4.32
Grade 3 vs 1	**0.001**	2.13	1.37-3.30	**<0.001**	3.04	2.02-4.58
LVI	**<0.001**	1.33	1.15-1.54	**<0.001**	1.36	1.20-1.53
NI	0.50	1.08	0.87-1.34	0.59	1.05	0.87-1.27

© 2023 Liu G et al. *JAMA Network Open.*

Number Of LNM	**<0.001**	1.05	1.05-1.06	**<0.001**	1.05	1.04-1.05
DM	**<0.001**	4.48	3.06-6.56	NA	NA	NA
Ki67	**0.002**	1.68	1.21-2.34	0.27	1.18	0.88-1.58
HR pos vs neg	**<0.001**	0.66	0.57-0.76	**0.004**	0.82	0.72-0.94
HER2 pos vs neg	0.09	0.88	0.77-1.02	0.05	0.89	0.79-1.00

Abbreviations: AC, apocrine carcinoma. BCS, breast-conserving surgery. DCIS, ductal carcinoma in situ. DFS, disease-free survival. DM, distant metastasis. HER2, human epidermal growth factor receptor-2. HR, hormone receptor. IDC, invasive ductal carcinoma. ILC, invasive lobular carcinoma. IMPC, invasive micropapillary carcinoma. LVI, lymphovascular invasion. LNM, lymph node metastases. M, mastectomy. MedC, medullary carcinoma. MIBC, microinvasive breast cancer. MPC, metaplastic carcinoma. MucC, mucinous carcinoma. NI, nerve invasion. OS, overall survival. OTIBC, other types of invasive breast cancer.

© 2023 Liu G et al. *JAMA Network Open.*

eTable 5. Characteristics of T1-2 IDC patients who underwent surgery before and after propensity score matching.

	Before Matching			After Matching		
	BCS	M	p	BCS	M	p
Number	1503	6879		1503	3006	
Sex (Female, %)	1502 (99.9)	6854 (99.6)	0.11	1502 (99.9)	2993 (99.6)	0.07
Age (mean (SD))	49.25 (10.98)	52.59 (10.37)	<0.001	49.25 (10.98)	49.84 (9.65)	0.06
Concurrent DCIS (%)	661 (44.0)	3184 (46.3)	0.11	661 (44.0)	1359 (45.2)	0.45
Invasive Size (median [IQR])	1.70 [1.20, 2.10]	2.00 [1.50, 2.60]	<0.001	1.70 [1.20, 2.10]	1.70 [1.20, 2.20]	0.43
Grade (%)			<0.001			0.38
1	154 (10.2)	426 (6.2)		154 (10.2)	272 (9.0)	
2	886 (58.9)	4091 (59.5)		886 (58.9)	1817 (60.4)	
3	463 (30.8)	2362 (34.3)		463 (30.8)	917 (30.5)	
LVI (%)	231 (15.4)	1468 (21.3)	<0.001	231(15.4)	463 (15.4)	1
NI (%)	129 (8.6)	622 (9.0)	0.61	129 (8.6)	248 (8.3)	0.75
Number Of LNM (median [IQR])	0.00 [0.00, 1.00]	0.00 [0.00, 2.00]	<0.001	0.00 [0.00, 1.00]	0.00 [0.00, 1.00]	0.39
Ki67 (median [IQR])	0.25 [0.10, 0.40]	0.25 [0.15, 0.40]	<0.001	0.25 [0.10, 0.40]	0.20 [0.15, 0.40]	0.67
HR (%)	1211 (80.6)	5181 (75.3)	<0.001	1211 (80.6)	2395 (79.7)	0.50
HER2 (%)	284 (19.9)	1978 (28.8)	<0.001	284 (18.9)	579 (19.3)	0.80

Abbreviations: BCS, breast-conserving surgery. DCIS, ductal carcinoma in situ. HER2, human epidermal growth factor receptor-2. HR, hormone receptor. IDC, invasive ductal carcinoma. LVI, lymphovascular invasion. LNM, lymph node metastases. M, mastectomy. NI, nerve invasion. OS, overall survival.

© 2023 Liu G et al. *JAMA Network Open.*

eFigure. Survival plot of T1-2 IDC patients underwent BCS and mastectomy (M) in the propensity-score matching cohort. A shows OS; B shows DFS.

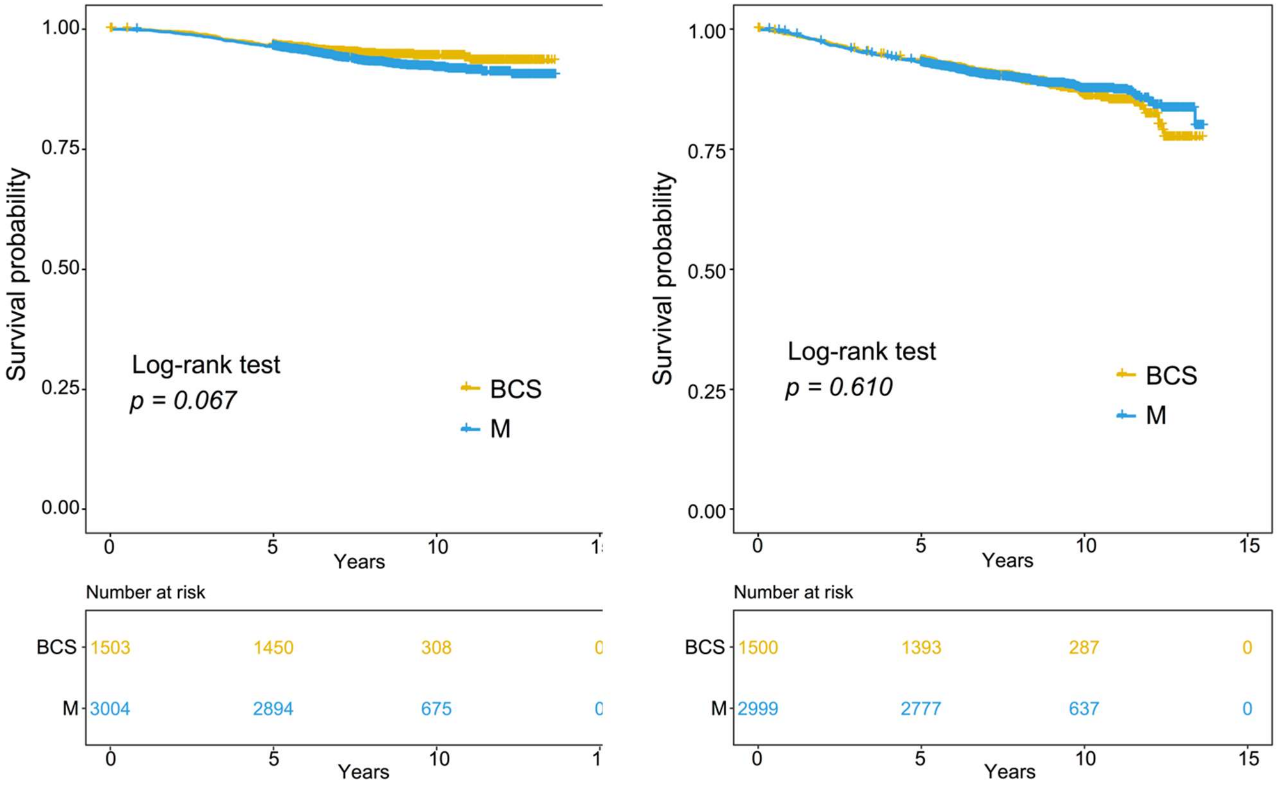

© 2023 Liu G et al. *JAMA Network Open*.
