# Supplementary material for: Clinical Features and Prognoses of Patients With Breast Cancer Who Underwent Surgery
Source: JAMA Netw Open. 2023 Aug 25;6(8):e2331078. doi: 10.1001/jamanetworkopen.2023.31078 (PMC10457722; doi:10.1001/jamanetworkopen.2023.31078)
Supplement: Supplement 2. — Data Sharing Statement [file jamanetwopen-e2331078-s002.pdf]

Data Sharing Statement

Liu. Clinical Features and Prognoses of Patients With Breast Cancer Who Underwent Surgery. *JAMA Netw Open*. Published August 25, 2023. doi:10.1001/jamanetworkopen.2023.31078

Data
Data available: Yes
Data types: Data (not involving human participants)
How to access data: Data are available upon reasonable request and requests must be sent to yidoctor99@126.com.
When available: With publication

Supporting Documents
Document types: None

Additional Information
Who can access the data: Data are available upon reasonable request.
Types of analyses: Data are available upon reasonable request.
Mechanisms of data availability: with investigator support
